# INPP5A/HLA-G1/IL-10/MMP-21 Axis in Progression of Esophageal Squamous Cell Carcinoma

**DOI:** 10.52547/ibj.3716

**Published:** 2022-10-04

**Authors:** Sima Ardalan Khales, Azadeh Aarabi, Mohammad Reza Abbaszadegan, Mohammad Mahdi Forghanifard

**Affiliations:** 1 Immunology Research Center, Mashhad University of Medical Sciences, Mashhad, Iran;; 2 Medical Genetics Research Center, Mashhad University of Medical Sciences, Mashhad, Iran;; 3 Department of Medical Genetics, Faculty of Medicine, Mashhad University of Medical Sciences, Mashhad, Iran;; 4 Department of Biology, Damghan Branch, Islamic Azad University, Damghan, Iran

**Keywords:** Esophageal squamous cell carcinoma, HLA-G antigen, INPP5A, Interleukin-10, MMP-21 protein

## Abstract

**Background:**

INPP5A is involved in different cellular events, including cell proliferation. Since *INPP5A*, *HLAG1*, *IL-10*, and *MMP-21* genes play fundamental roles in ESCC tumorigenesis, we aimed in this study to clarify the possible interplay of these genes and explore the potential of these chemistries as a predictor marker for diagnosis in ESCC disease.

**Methods:**

Gene expression analysis of *INPP5A*, *HLAG*-*1*, *IL-10*, and *MMP-21* was performed using relative comparative real-time PCR in 56 ESCCs compared to their margin normal tissues. Immunohistochemical staining was accomplished for INPP5A in ESCCs. Analysis of ROC curves and the AUC were applied to evaluate the diagnostic capability of the candidate genes.

**Results:**

High levels of *HLA-G1*, *MMP-21*, and *IL-10* were detected in nearly 23.2%, 62.5%, and 53.5% of ESCCs compared to the normal tissues, respectively, whereas *INPP5A* underexpression was detected in 19.6% of ESCCs, which all tested genes indicated significant correlations with each other. The protein expression level of INPP5A in ESCC tissues was significantly lower than that of the non-tumor esophageal tissues (*p* = 0.001). Interestingly, the concomitant expression of the *INPP5A/HLA-G1, INPP5A*/*MMP-21, INPP5A*/*IL-10, HLA-G1*/*MMP-21, HLA-G1*/*IL-10*, and *MMP*-21/IL-10 was significantly correlated with several clinicopathological variables. *INPP5A*, *HLA-G1*, *MMP-21*, and *IL-10* showed to be the most appropriate candidates to discriminate tumor/non-tumor groups due to the total AUCs of all combinations (>60%).

**Conclusion:**

Our results represent a new regulatory axis containing *INPP5A/HLAG-1/IL-10/MMP-21* markers in ESCC development and may provide novel insight into the mechanism of immune evasion mediated by the *INPP5A/HLAG-1/IL-10/MMP-21* regulatory network in the disease.

## INTRODUCTION

Esophageal cancer, as one of the aggressive malignancies with high mortality rate, needs relatively a better diagnostic and therapeutic improvements^[^^1^^]^. ESCC is considered the most frequent histological subtype of EC (>90%) globally and may be caused due to its drastically invasive nature and poor prognosis^[^^2^^]^. Inadequate understanding of molecular and cellular mechanisms limits the improvement of prognosis and therapy of the disease. Thus, the identification of novel therapeutic targets is urgent to overcome such failures.

Inositol phosphates, the highly conserved eukaryotic signaling molecules, has regulatory function in various biological processes, such as embryonic development, mRNA export from the nucleus, signaling transduction pathways, and stress responses^[^^3^^]^. The INPP5A belongs to a ubiquitous family of the phosphoinositide 5-phosphatases, functioning predominantly as a signal-terminating enzyme associated with various cellular processes like cell proliferation^[^^4^^]^. 

The cancer immune-editing process, an essential host protection response, consists of three sequential phases, including elimination, equilibrium, and escape^[^^5^^]^. Interestingly, cancer cells interfere with these phases through an aberrant expression of the nonclassical HLA-G during the development of an overt malignancy^[^^5^^]^. The human HLA-G is a member of the MHC-Ib family, which was first discovered in extravillous cytotrophoblasts^[^^6^^]^ and expressed in several pathological situations, such as cancers and inflammatory and autoimmune diseases^[^^7^^]^. As a double-edged sword, the immunoregulatory cytokine IL-10 is implicated in either cancer promotion^[^^8^^]^ or inhibition^[^^9^^]^, depending on the context during carcinogenesis. Moreover, IL-10 production has been restricted to immune cells (e.g. monocytes and granulocytes), nonimmune cells (e.g. keratinocytes and epithelial cells), and cancer cells^[^^10^^]^. Besides, IL-10 induces cancer cell proliferation and metastasis through immunosuppression^[^^11^^]^.

MMP are dominant mediators of cell dissemination and migration through the degradation of the basement membrane and extracellular matrix components^[^^12^^]^. Notably, the key hallmarks of cancer, including spreading, invasion, metastasis, and angiogenesis, are tightly correlated with MMPs by degrading different cell adhesion molecules and modulating cell-cell and cell-matrix interactions^[13]^. MMPs are significantly upregulated in cancers such as breast and ovarian cancers, as well as head and neck squamous cell carcinomas^[^^14^^]^. While MMP-21 is normally implicated in embryogenesis, its abnormal function was detected in different malignancies, especially ESCC^[^^15^^,^^16^^]^. MMP-21 expression is significantly higher in the lymph node metastatic oral squamous cell carcinoma than the primary tumors, which is correlated with poor overall survival^[^^17^^]^. Similarly, upregulation of MMP-21 is found in well- and moderately differentiated pancreatic cancer cells^[^^18^^]^. It has been also reported that the MMP-21 expression is considerably associated with the TNM stage, LNM, and overall survival in ESCC^[^^19^^]^. 

Based on the integral role of each gene in different cancers, we took the opportunity to focus on novel potential regulatory crosstalk among the *INPP5A, HLA-G1, IL-10, *and *MMP-21* in ESCC. To this end, we evaluated clinicopathological relevance of the genes in the disease and offered reliable predictor markers for ESCC diagnosis.

## MATERIALS AND METHODS

**Patients and tissue specimens**

In this study, 56 surgical ESCC tissues and paired adjacent normal tissues were acquired from Omid Hospital of Mashhad University of Medical Sciences (Mashhad, Iran). All the ESCC specimens were confirmed based on pathological diagnosis, and none of the patients declared to have a history of chemotherapy, radiotherapy, or biological therapy before undergoing surgery. The ESCC stage was classified according to the TNM system of the Union International Cancer^[^^20^^]^.

**RNA extraction, cDNA synthesis, and qRT-PCR**

The mRNA expression levels of *INPP5A*,* HLA-G1*,* IL-10*, and *MMP-21* genes were conducted using qRT-PCR. Total RNA was extracted from tumor and normal tissues of esophageal specimens using RNeasy Mini Kit (Qiagen, Germany). The RNA samples were treated with DNase I (Thermo Fisher Scientific, Waltham, MA, USA) to eliminate any DNA contamination. Following the completion of cDNA synthesis (oligo dT method) using the PrimeScript™ 1st Strand cDNA Synthesis Kit (Takara, Otsu, Japan), qRT-PCR was carried out with gene-specific primer pairs (Microgene, Korea; Table 1) and SYBR-Green Master (Takara Bio, Japan) and analyzed on a Roche LightCycler® 96 System (Germany) according to the manufacturer’s instructions. Relative transcript quantities were determined using the Log2 (fold change) method and normalized with *GAPDH* as the endogenous reference gene. The experiments were carried out in triplicates.

**Table 1:** Gene-specific primer sets used in qRT-PCR

**Genes**	**Forward primer sequence**	**Reverse primer sequence**	**Annealing, T (°C)**
*GAPDH *	GGAAGGTGAAGGTCGGAGTCA	GTCATTGATGGCAACAATATCCACT	60
*INPP5A *	TTGCAGACTGTGCCTTTGAC	AAACCTTCTCGAATCGCTGA	58
*HLA-G1*	CTGGTTGTCCTTGCAGCTGTAG	CCTTTTCAATCTGAGCTCTTCTTTCT	60
*IL-10*	AACCAAGACCCAGACATCAAGG	CATTCTTCACCTGCTCCACG	60
*MMP-21*	TCGACATCAAGCTGGGCTTT	ACCTTGAGAAGGCTGATGCC	60

**IHC staining**

The protein expression levels of INPP5A in esophageal cancerous tissues and adjacent non-cancerous tissues were evaluated using IHC. FFPE of 30 paired ESCC tumor tissues and non-cancerous tissues were retrospectively gathered. Briefly, 4–5 μm FFPE tissue sections were deparaffinized, rehydrated, and subjected to antigen retrieval as described before^[^^21^^]^. Each section was blocked with 100–400 μl of bovine serum albumin solution (RE7102, Thermo Fisher Scientific) at room temperature for 1 h. The tissue slides were then incubated with the rabbit anti-INPP5A antibody, (ab118418, Abcam) at a volume ratio of 1:50 at 4 °C for 1 h. Subsequently, the slides were incubated with HRP-conjugated secondary antibody (#8114, Cell Signaling Technology, Danvers, MA, USA) at room temperature for 30 min and then stained with diaminobenzidine 3,3′-tetrahydrochloride detection kit (RE7105, Leica- NovoCastra, Newcastle, UK) according to the manufacturer’s protocol. Finally, the slides were counterstained with Mayer’s hematoxylin (100–400 μl per section) for 10 min. For the INPP5A, human ESCC tissues with and without primary antibodies were used as positive and negative controls in each batch of IHC staining.

**Validation of IHC staining**

Immunohistochemical evaluation was carried out by two pathologists based on the following criteria: staining intensity: 0 (negative), 1 (weak), 2 (moderate), and 3 (strong), as well as percentage of positive cells: 0 (<5%), 1 (5-25%), 2 (26-50%), 3 (51-75%), 4 (76-100%). The final IHC score was determined by multiplying the staining intensity score by the score of positive cells ranging from 0 to 12. The intensity score was calculated for each section applying the following formula: Percentage of INPP5A positive cells (P) × intensity (I)^[^^22^^]^.

**Diagnostic analysis**

ROC curves and the AUC were applied to assess the diagnostic effectiveness of candidate genes for the discrimination between tumor and non-tumor samples of ESCC by calculating specificity and sensitivity for probable cut-off values of the candidate genes. Sensitivity and specificity levels were achieved according to Youden's index (sensitivity + 100% -specificity)^[^^23^^]^. 

**Bioinformatics analysis**

The biological database, GeneMANIA (http://www. genemania.org), was employed to predict a large set of functional interaction networks, such as co-expression, genetic and protein interactions, gene co-location, pathways, and protein domain similarities. The protein correlation networks closely relating to the co-expression of *INPP5A*, *HLAG1*, *IL-10*, and *MMP-21* were determined.

**Statistical analysis**

All statistical tests were conducted using SPSS 19.0 (SPSS Inc., Chicago, IL, USA), and figures were generated via GraphPad Prism 5.0 (GraphPad Software, Inc., La Jolla, CA, USA) software. The correlation between the *INPP5A*, *HLA-G1*, *IL-10*, and *MMP-21* genes was calculated using Pearson’s correlation and χ2 or Fisher exact tests. The statistical association between the expression levels of the selected genes and different clinicopathological properties was evaluated using the t-test and ANOVA. A *p* < 0.05 was regarded as statistically significant.

## RESULTS

**Demographic and clinicopathological parameters of the studied samples**

Of 56 ESCC patients, 32 (57.1%) were males, and 24 (42.9%) were females (mean age ± SD: 62.85 ± 13.24, range 32–85 years). Of these -56 samples, 10 (17.8%) cases were poorly differentiated, 34 (60.7%) cases were moderately differentiated, and 12 (21.4%) cases were well differentiated. Moreover, 45 (80%) cases were presented with invaded cancer cells into the adventitia (T3), and 11 (19.6%) cases without invaded cancer cells (T1 and T2); 25 (44.6%) cases revealed LNM, and 31 (55.4%) without LNM. Regarding the TNM staging criteria, 35 (62.5%) cases were stage I and II, and 21 (37.5%) were stage III (Table 2). After evaluating the mRNA expression of 56 tumor samples using qRT-PCR, 30 samples showing distinct pattern of gene expression, were selected for IHC analysis. The median age of these 30 patients was 56.5 years (range 30-83 years), with an equal gender distribution of 17 males and 13 females. Tumor sizes were between 2 and 9 cm (mean size ± SD: 4.84 ± 2.41). Also, 14 (46.6%) patients were featured as moderate differentiation of ESCC, while 8 (26.6%) and 8 (26.6%) patients were featured as poor and well differentiation, respectively. T3 tumor depth of invasion was found in the majority of patients (73.3%). T1 and T2 values were seen in 6.6% and 20% of the patients, respectively. 15 of the tumors had no lymph node metastasis. IIa/IIb stage distributions of ESCC samples were 18 (60%), and 12 (40%) samples were stage III (Table 3). The ESCC patients' clinicopathological parameters and expression profiles of *INPP5A, HLA-G1, IL-10,* and *MMP-21* are presented in Table 2.

**Correlation between** ***INPP5A*** **expression and clinicopathological parameters**

**Fig. 1:**
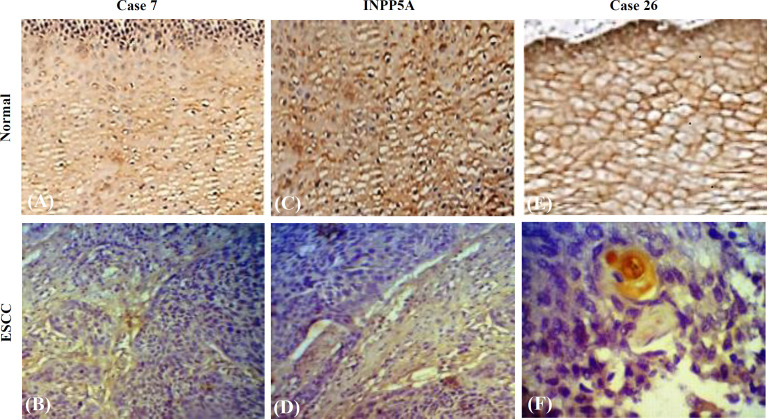
Immunohistochemistry staining patterns of INPP5A protein expression in normal and ESCC tissues in different cases. (A, C, and E) INPP5A expression in the normal esophageal epithelium (cases 7, 18, and 26); (B, D, F) weak expression of INPP5A in ESCC tissues (cases 7, 18, and 26). (A, B, C, D, original magnification ×200; E, F original magnification ×400)

The mRNA and protein expression levels of INPP5A were assessed using RT-qPCR and IHC analysis, respectively. The mRNA expression level of *INPP5A* was significantly lower in ESCC tissues compared to the matched adjacent non-tumor esophageal tissues (mRNA expression, *p* = 0.018). Low level of *INPP5A* mRNA expression was significantly associated with gender (*p* = 0.024) and location (*p* < 0.001). In addition, the *INPP5A* mRNA expression was inversely associated with not only the high grade of tumor differentiation (*p* = 0.017) but also tumor invasion (*p* < 0.001). IHC analysis was used to detect INPP5A expression in 30 paired ESCC specimens and the matched normal tissues. Remarkably, the IHC results verified that the protein expression of INPP5A in ESCC tissues was lower than that of normal samples (*p* = 0.001). In all paraffin-embedded ESCC tissues, weak expression of the INPP5A was observed in 50% samples (15/30), moderate expression in 13% samples (4/30), and strong expression in 11 samples (36%) (Fig. 1). The associations between INPP5A protein expression and other clinical variables in ESCC patients indicated that the underexpression of the INPP5A protein was significantly correlated with age (*p* = 0.055) and tumor size (*p* = 0.037). No significant association was detected between the INPP5A protein expression and other clinicopathological parameters (Table 3). 

**Table 2:** Clinicopathological parameters of the study population and expression profiles of *INPP5A*, *HLA-G1*, *IL-10*, and *MMP-21*

**Parameters**	**No.**		***INPP5A*** **expression**			***HLA-G1*** **expression**			***MMP-21*** **expression**			***IL-10*** ** expression**	
			**-/**	**-/**		**-/**	**-/**		**-/**	**-/**		**-/**	**-/**
**Gender**													
Male	32		30	2		24	8		13	19		15	17
Female	24		21	3		19	5		9	15		8	13
													
**Grade of differentiation**													
P.D.	10		7	3		8	2		5	5		2	6
M.D.	34		33	1		25	9		13	21		15	19
W.D.	12		11	1		10	2		4	8		7	5
													
**Tumor location**													
Lower	23		20	3		21	2		10	13		10	13
Middle	32		31	1		21	11		12	20		15	17
Upper	1		0	1		1	0		0	1		1	0
													
**Depth of tumor invasion**													
T1	3		1	2		3	0		1	2		1	2
T2	8		8	0		5	3		2	6		5	3
T3	45		42	3		35	10		19	26		20	25
													
**LNM**													
No metastasis	31		27	4		23	8		12	19		11	20
Node metastasis	25		24	1		20	5		10	15		15	10
													
**Stage of tumor progression**													
I	1		1	0		1	0		1	0		0	1
IIa	29		26	3		21	8		11	18		11	18
IIb	5		3	2		3	2		0	5		3	2
III	21		21	0		18	3		10	11		12	9
IV	0		0	0		0	0		0	0		0	0

P.D., poorly differentiated; M.D., moderately differentiated; W.D., well differentiated; , overexpression; , underexpression ; -, normal

**Table 3:** Correlation of INPP5A protein expression with different clinicopathological parameters of ESCC patients

**Parameters**		**Protein**				***p *** **value**
		**Negative**	**Low**	**High**		
**Gender**						
Male		17	9	6		0.357
Female		13	6	5		
						
**Grade of differentiation**						
P.D.		8	2	0		0.466
M.D.		14	11	9		
W.D.		8	1	3		
						
**Tumor location**						
Lower		14	4	5		0.438
Middle		15	10	7		
Upper		1	0	0		
						
**Depth of tumor invasion**						
T1		2	0		1	0.795
T2		6	1		1	
T3		22	13		10	
						
**LNM**		15	7		9	0.174
No metastasis		15	7		3	
Node metastasis		0	0		1	
						
**Stage of tumor progression**						
I		0	0		1	0.872
IIa		14	14		8	
IIb		4	4		0	
III		12	12		3	
IV		0	0		0	

P.D., poorly differentiated; M.D., moderately differentiated; W.D., well differentiated

**Correlation between** ***INPP5A, HLA-G1, IL-10*** **, and** ***MMP-21*** **genes in ESCC**

**Fig. 2:**
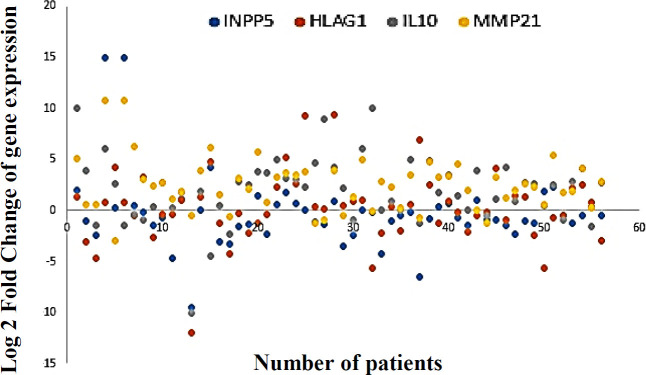
The scatter plot representing the gene expression distribution of *INPP5A*,* HLA-G1*,* IL-10*, and *MMP-21* in ESCC patients. Relative mRNA expression more than twofold in tumor tissue is defined as overexpression, less than twofold as underexpression, and the range fold change between ± 2 is considered as normal expression.

**Fig. 3:**
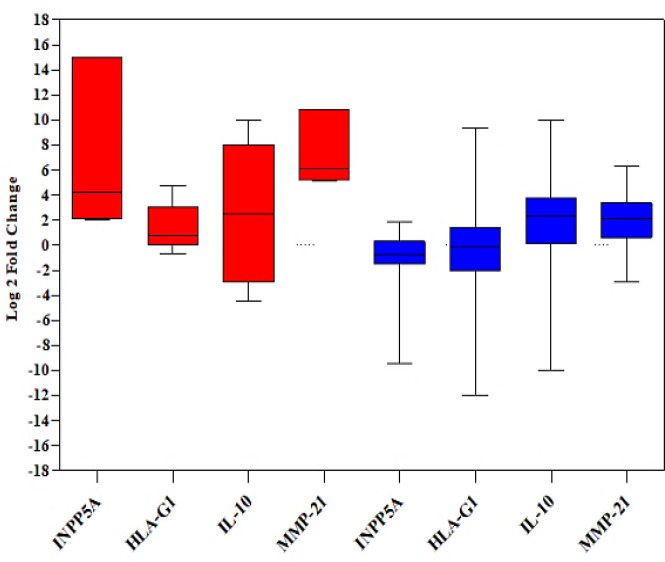
The box plot shows the fold change in expression of the genes. The samples were divided into two groups according to the gene expression patterns (overexpression and normal/ underexpression), and the expression levels of both groups were compared. Red and blue show upregulation and downregulation of genes

The mRNA expression of the *HLA-G1*,* IL-10*, and *MMP-21*, as well as the potential correlation between the expression level of *INPP5A* and the selected genes, were evaluated in ESCC patients. The gene expression patterns of the *INPP5A, HLA-G1, IL-10*, and *MMP-21* in ESCC specimens are exhibited as a scatter plot in Figure 2. Interestingly, all examined genes indicated significant correlations with each other (Table 4). It means that the low level of *INPP5A* expression was associated with the overexpression of *HLA-G1*,* IL-10*, and *MMP-21* in ESCCs compared to the non-cancerous tissues (Fig. 3).

**Correlation of concomitant expression of** ***INPP5A, HLA-G1, IL-10,*** **and** ***MMP-21*** **in different clinicopathologic parameters**

Having analyzed data, we found a significant correlation between *INPP5A* and *MMP-21* mRNA expression in high-grade (grade 3) than that of low-grade (grades 1 and 2) states of the esophageal tissue samples (*p* < 0.05), as well as LNM (*p* < 0.05; Table 5). Furthermore, the concomitant expression patterns of *INPP5A/HLA-G1, INPP5A/IL-10*, and *INPP5A/MMP-21* were significantly associated with each other in tumors with invasion to adventitia (T3) (*p* = 0.006, *p* = 0.001 and *p* = 0.001, respectively). Importantly, the association of concomitant expression of *INPP5A/IL-10 *and *INPP5A/MMP-21* in tumors with advanced stage (III) compared to the early stages (I and II) was statistically significant (*p* < 0.001), suggesting that the co-expression pattern of *INPP5A* with *HLA-G1, IL-10,* and *MMP-21* are dependent on these aggressive tumor features. 

**Association of concomitant expression of HLA-G1 with IL-10, and MMP-21 in different clinicopathologic parameters**

Regarding the tumor depth invasion in the esophageal wall (T staging), concomitant overexpression of *HLA-G1* with *IL-10* (*p* = 0.048) and *MMP-21 *(*p* = 0.043) was observed in ESCC patients with T3 depth of invasion. Moreover, the concomitant *HLA-G1* and *IL-10* overexpression was found to be significantly associated with lymph node status (*p* = 0.018; Table 5). Also, the concomitant overexpression of IL-10 and *MMP-21* was observed in tumor samples with stage III (*p* = 0.027). As shown in Table 5, co-overexpression of *HLA-G1/IL-10* and *HLA-G1/MMP-21* genes was correlated with an increased ESCC stage, and *HLA-G1* and *IL-10* (*p* = 0.052) were remarkably correlated with the stage III (Table 5). 

**Diagnostic analysis**

**Fig. 4:**
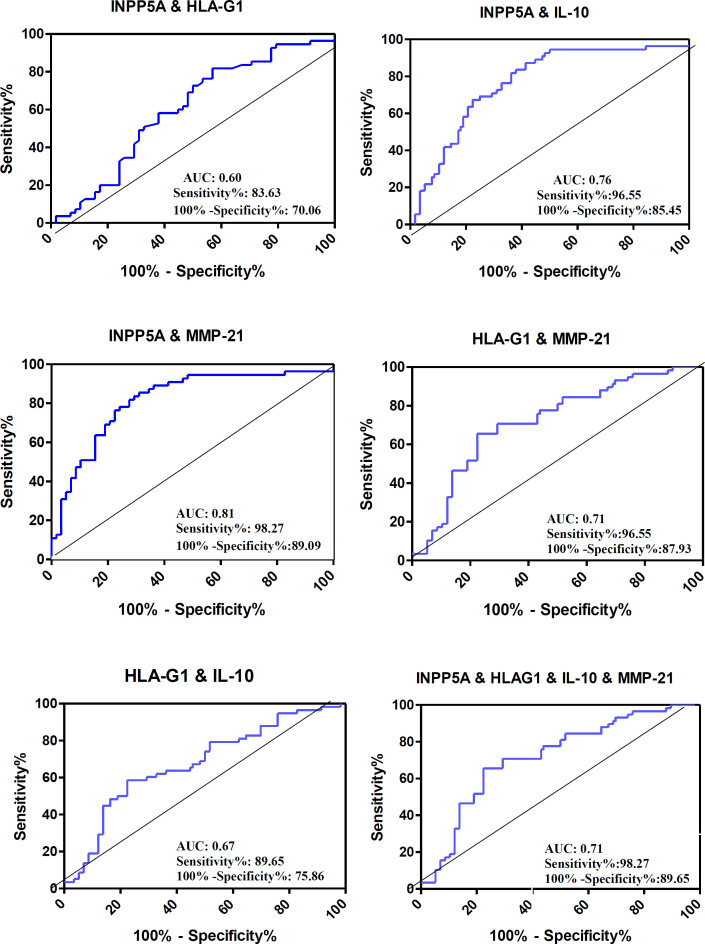
ROC curve analysis of the combination of candidate genes. The AUC, sensitivity, and specificity are shown in each subgraph. Combination ROC curve of (A) *INPP5A* *+* *HLA-G1*, (B) *INPP5A* *+* *IL-10*, (C) *INPP5A* *+* *MMP-21*, (D) *HLA-G1* *+* *IL-10*, (E) *HLA-G1* *+* *MMP-21*, and (F) *INPP5A* *+* *HLA-G1* *+* *IL-10* *+ MMP-21*

For evaluating the diagnostic capability of the candidate genes to discriminate tumor and non-tumor states, we conducted multivariate ROC analyses for combinations of mRNA candidates. Remarkably, total AUCs of all combinations were significant and >60% (Fig. 4). A detailed analysis of each ROC curve includes the combination of the *INPP5A* + *HLA-G1* (AUC = 0.60; *p* = 0.05; sensitivity, 83.63; and specificity, 70.06), the *INPP5A* + *IL-10* (AUC = 0.76; *p* < 0.0001; sensitivity, 96.55; and specificity, 85.45), the *INPP5A* + *MMP-21* (AUC = 0.81; *p* < 0.0001; sensitivity, 98.27; and specificity, 89.09), the *HLA-G1 *+ *MMP-21* (AUC = 0.71; *p* < 0.0001; sensitivity, 96.55; and specificity, 87.93), the *HLA-G1* + *IL-10* (AUC = 0.67; sensitivity, 89.65; and specificity, 75.86), and the *INPP5A *+ *HLA-G1* + *IL-10* + *MMP-21* (AUC, 0.71; *p *< 0.0001; sensitivity, 98.27; and specificity, 89.65) gene expression, which are illustrated in each subgraph in Figure 4.

**Table 4:** Correlation between *INPP5A*,* HLA-G1*,* IL-10*, and *MMP-21* mRNA expression in ESCC patients

**Gene**	***INPP5A***	***HLA-G1***	***MMP-21***	***IL-10***
***INPP5A***				
Pearson Correlation	1	0.373^*^	0.730^**^	0.462^*^
Sig. (2-tailed)		0.036	0.000	0.012
				
***HLA-G1***				
Pearson Correlation	0.373^*^	1	0.528^**^	0.332^*^
Sig. (2-tailed)	0.036		0.006	0.032
				
***MMP-21***				
Pearson Correlation	0.730^**^	0.528^**^	1	0.924^**^
Sig. (2-tailed)	0.000	0.006		0.000
				
***IL-10***				
Pearson Correlation	0.462^*^	0.332^*^	0.924^**^	1
Sig. (2-tailed)	0.012	0.032	0.000	

^*^Correlation is significant at the 0.05 level; ^**^correlation is significant at the 0.01 level (2-tailed)

**Protein-protein interaction network analysis**

**Fig. 5:**
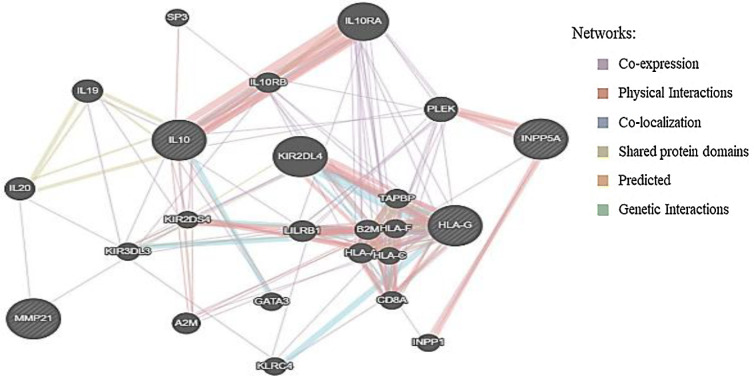
Protein-protein interaction networks generated using the GeneMANIA database

This analysis was carried out using the GeneMANIA database to elucidate the relationships among co-expression, pathways, co-location, prediction, and the shared protein domains. The results exhibited the co-expression of *INPP5A*,* HLAG1*,* IL-10*, and *MMP-21* with several genes, such as *IL-19*, *1L-20*, *IL10RA*, *IL10RB*,* GATA3*,* INPP1*,* HLA-A*,* HLA-F*,* TAPBP*,* B2M*,* LILRB1*,* PLEK*,* CD8A*,* KLRC4*,* KIR2DS4*, and *KIR3DL3* (purple lines in Fig. 5). Other interactions are also presented in Figure 5. These findings verified the biological relevance among *INPP5A*,* HLAG1*,* IL-10*, and *MMP-21* genes.

## DISCUSSION

Inositol signaling pathways are implicated in membrane trafficking, intracellular calcium (Ca^2+^) release, ion channel function, chemotaxis, and gene regulation^[^^3^^]^. It is well established that dysregulation of inositol 1,4,5-trisphosphate-Ca^2+^ signaling pathway results in abnormal changes in intracellular Ca^2+^ concentration, which has been detected in severe pathological processes, such as cancer^[^^24^^]^, Alzheimer’s disease^[^^25^^]^, primary myelofibrosis^[^^26^^]^, and amyotrophic lateral sclerosis^[^^27^^]^, as well as physiological processes including cancer cell proliferation, migration, metastasis, invasive pathways, and inflammation^[^^24^^]^. 

The majority of investigations have focused primarily on the alteration of individual gene expression in ESCC tumorigenesis while overlooking coordinated transcriptional changes of tumor genes. Herein, we presented the significance of examining gene co-expression networks in addressing ESCC tumorigenesis. We also remarkably found higher levels of *HLA-G1*,* MMP-21*, and *IL-10* gene expression in ESCCs against the normal tissues, while underexpression of *INPP5A* was detected in ESCCs. All tested genes indicated significant correlations with each other. Interestingly, the concomitant expression of the selected genes was significantly correlated with the several clinicopathological characteristics.

**Table 5:** Correlations between the ESCC patients' clinicopathological parameters and co-expression profiles of *INPP5A*,* HLA-G1*,* IL-10*, and* MMP-21*

**Factor **		**Co-expression of the genes (** ***p value*** **)**					
		***INPP5A*** **&** ***HLA-G1***	***INPP5A*** **&** ***IL-10***	***INPP5A*** **&** ***MMP-21***	***HLA-G1*** **& ** ***IL-10***	***HLA-G1*** **& ** ***MMP-21***	***IL-10*** ** &** ***MMP-21***
Grade of differentiation T3							
Pearson correlation		0.404^**^	0.477^**^	0.478^**^	0.296^*^	0.303^*^	-
Sig. (two-tailed)		0.006	0.001	0.001	0.048	0.043	-
		
Depth of tumor invasion P.D							
Pearson correlation		-	-	0.935^**^	-	-	-
Sig. (two-tailed)		-	-	0.000	-	-	-
		
Lymph node metastasis node metastasis							
Pearson correlation		-	-	0.809^**^	0.473^*^	-	-
Sig. (two-tailed)		-	-	0.000	0.017	-	-
		
Stage of tumor progression III							
Pearson correlation		-	0.799^**^	0.572^**^	0.582^**^	-	0.483^*^
Sig. (two-tailed)		-	0.000	0.007	0.006	-	0.027

^*^Correlation is significant at the 0.05 level; ^**^correlation is significant at the 0.01 level (2-tailed)

*In vitro* and *in vivo* investigations have demonstrated that INPP5A downregulation leads to cellular transformation and cancer development, suggesting a vital role for *INPP5A* as a tumor suppressor gene through preventing cancer cell proliferation^[^^28^^]^. The depletion of INPP5A, as well as intracellular Ca^2+^ oscillations and transformation in cell culture, contributes to cancer development^[^^28^^-^^30^^]^. Deletion of a region encoding INPP5A on chromosome 10q26 is reported in cutaneous squamous cell carcinoma^[^^31^^]^ and brain tumors^[^^32^^]^. A reduction in INPP5A expression level emerges to be a prior event in the SCC development, and loss of its expression is found in the earliest stage of SCC, and actinic keratosis^[^^31^^]^. A progressive reduction in INPP5A expression level plays a significant role in the progression of the localized to metastatic oropharyngeal SCC and cutaneous squamous cell carcinoma, which associates with invasive tumor behavior and poor clinical outcomes^[^^31^^]^. To this end, we assessed INPP5A protein expression level in ESCC patients. The IHC findings detected a decline in INPP5A protein level in 50% of the examined ESCC samples compared with that of the normal matched tissues. Significantly, the low level of INPP5A protein expression was associated with age and tumor size. Furthermore, the decreased mRNA expression level of *INPP5A* was not only inversely correlated with the histologic grade and depth of invasion but also with patients’ sex, location, and tumor size. As a consequence, our data collectively uncover the crucial regulatory role of *INPP5A* as a novel tumor suppressor gene in ESCC. In addition, we evaluated the correlation of *HLA-G1, IL-10, *and *MMP-21* with *INPP5A* in ESCC. In this study, we demonstrated that the concomitant expression of *INPP5A/MMP-21* had a significant link to each other in patients with high-grade tumors with LNM. Besides, there were significant correlations between the concomitant expression profiles of *INPP5A/HLA-G1, INPP5A/IL-10*, and *INPP5A/MMP-21* in the patients with invaded cancer cells into the adventitia. More specifically, our statistical analysis elucidated that the expression patterns of *INPP5A, IL-10*, and *MMP-21* were correlated with each other in the advanced stages, as same as the expression patterns of *INPP5A, HLA-G, IL-10*, and *MMP-21*. Therefore, our observations propose that the regulatory networks among these genes may facilitate our understanding of this part of the complex molecular mechanisms governing the ESCC progression and metastasis. 

It is well-established that the TNM staging system has been considered as the primary indicator for predicting prognosis and even the infrastructure for guiding the treatment. However, the TNM system has its restrictions for ESCC due to broadly varied clinical prognosis in tumors with the same pathological stage^[^^33^^]^. Hence, identifying a reliable and precise predictor tumor-related marker for ESCC patients is urgent. At present, several proteins such as carcinoembryonic antigen, carbohydrate antigen 19–9, cytokeratin 19 fragment antigen 21–1, squamous cell carcinoma antigen, and carbohydrate antigen 72–4 are generally applied as a diagnosis, as well as prognostic predictors for various cancers^[^^34^^-^^36^^]^, including ESCC^[^^37^^,^^38^^]^. However, there is disagreement on which tumor biomarker is the best predictor for prognosis and diagnostic in ESCC patients. In medical research, the utilization of multiple markers in combination considerably increases the accuracy of diagnostic tests^[^^39^^]^. Herein, the AUC values were calculated for the ESCCs against non-tumor samples through multivariate ROC analyses for combinations of candidate markers. Our ROC curves remarkably indicated significantly greater AUC value than 60%, which can serve as predictors for diagnostic in ESCC. Therefore, our results showed that these crosstalk networks maybe powerful diagnostic capabilities in discriminating between tumor and non-tumor samples.

Cancer immune evasion can be due to functional and structural modifications of HLA-class I molecules and/or the detrimental activity of immunosuppressive cytokines, including IL-10, which is recognized as one of the hallmarks of cancer^[^^40^^]^. Among the cancer-produced immunosuppressive cytokines, IL-10 is responsible for the overexpression of HLA-G, which is implicated in the immune escape^[^^41^^]^. On the other hand, the HLA-G expression itself has been shown to stimulate the T-helper cell type-2 (Th2)-skewing condition and produce the IL-10. Activation of HLA-G expression by IL-10 is indicated in melanoma cells, thereby contributing to the tumor escape from immunosurveillance^[^^42^^]^. All these mechanisms can meaningfully change anticancer immune responses, leading to cancer growth and progression through the blocking of immunity system and triggering tolerance to cancer. Consistently with these observations, our expression analyses indicated that the expression pattern of the *HLA-G* and *IL-10* was considerably associated with each other in the higher surgical stage (stage III) of the disease. Moreover, the concomitant overexpression of the genes was associated with T3 and LNM of ESCC patients. Consequently, there may be a positive feedback amplification loop between IL-10 and HLA-G expression in ESCC. Additionally, the HLA-G expression is also considered as the cancer-driving force with aggressiveness and metastasis potential in patients and preclinical models through the mechanism of overexpression of cancer metastasis-related factors, including MMPs^[^^42^^]^. Several studies have revealed a close relationship between HLA-G and MMPs expression^[^^43^^,^^44^^]^. The high MMP-14 expression in the HLA-G-positive first-trimester invasive trophoblasts was also addressed^[^^43^^]^. Besides, the characterization of the molecular mechanisms whereby HLA-G is implicated in cancer invasion and metastasis indicates that MMP-15 overexpression is in compliance with HLA-G expression, and a notable association was also detected between HLA-G and MMP-15 expression in patients with ovarian cancer^[^^44^^]^. Moreover, we have previously provided evidence for the upregulation of *MMP-21* in ESCC patients, which was correlated with advanced stages and tumor invasion depth^[^^16^^]^; thus, it facilitates the aggressive pattern of these lesions. Consistently with these outcomes, our expression analyses in ESCC strongly underline the interdependence of the HLA-G1 and MMPs in the invasive behavior of tumors. Indeed, our finding demonstrated the significant correlation between concomitant expression of *HLA-G1* and *MMP-21* with the invasion of the tumor to adventitia (T3) in ESCCs. Interestingly, the *in silico* observations confirmed the results of our functional in ESCC patients. The *in silico* data indicated the co-expression of *INPP5A*,* HLAG1*, *IL-10*, and *MMP-21* with different genes, including *IL-19*,* 1L-20*,* IL10RA*, *IL10RB*,* GATA3*,* INPP1, HLA-A*,* HLA-F*,* TAPBP*, *B2M*,* LILRB1*,* PLEK*,* CD8A*,* KLRC4*,* KIR2DS4*, and *KIR3DL3*, which further supports our findings. 

**Fig. 6:**
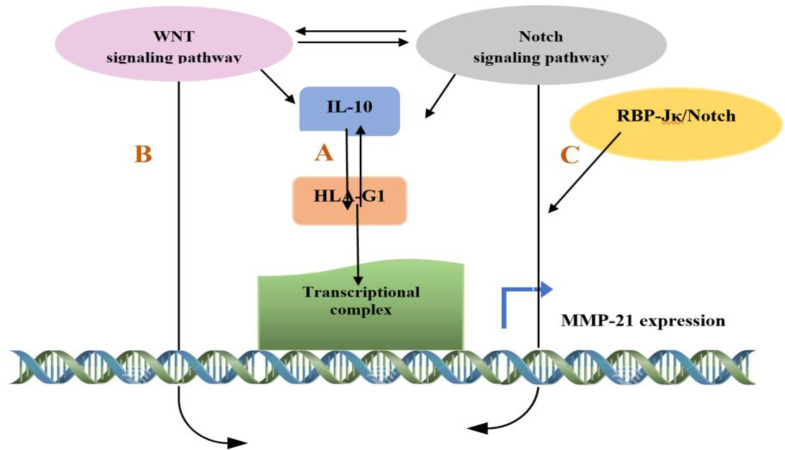
The probable crosstalk between HLA-G1, IL-10, and MMP-21 and signaling pathways in ESCC. (A) A positive feedback regulation between IL-10 and HLA-G (B) MMP-21 interacting with the TGF-beta /smad pathway through the putative binding sites of the LEF-1/Tcf-4 regulatory element at its promoter. (C) The regulation of MMP-21 expression by the Notch signaling pathway through the RBP-Jκ/Notch motifs in the MMP-21 promoter

While the Wnt/beta-catenin and Notch signaling pathways are critical during embryonic development and tissue homeostasis, both are also essential during ESCC progression^[^^45^^-^^47^^]^. MMP-21 is a potential Wnt/ beta-catenin transduction pathway target, which interacts with the *transforming growth factor*-beta (TGF-beta)/Smad pathway owing to the existence of the putative binding sites of the LEF-1/Tcf-4 regulatory element at its promoter^[^^48^^]^. Similarly, the MMP-21 expression is regulated uniquely by the Notch signaling pathway through the presence of the highly unusual RBP-Jκ/Notch motifs in the upstream of the transcription start site of the MMP-21 promoter^[^^49^^]^. Furthermore, Wnt/beta-catenin and Notch signaling pathways are the principal regulators of cancer immune responses^[^^50^^]^. Based on different models of murine cancer, numerous reports have shown that the upregulation of Wnt ligands produce IL-10 in tumors program dendritic cells, which contributes to the immune inhibition through promoting T regulatory cell responses^[^^51^^,^^52^^]^. Indeed, this mechanism is due to the activation of Wnt/beta-catenin signaling, which results in elevating immune-regulatory molecules expression, particularly IL-10 in the tumor microenvironment dendritic cells^[^^53^^]^. Furthermore, Notch signaling stimulates IL-10 production following interaction of Notch with their ligands like DLL-1 and DLL-4^[^^54^^]^. In addition to positive feedback regulation between IL-10 and HLA-G, the elevation of IL-10 is dependent on the activation of beta-catenin/ Tcf- 4 and Notch pathways (Fig. 6). Consequently, the crosstalk of Wnt/Notch signaling cascades leads to the activation of the downstream target genes, such as *MMP-21*,* HLA-G*, and *IL-10*.

Several studies have shown a molecular linkage between the Wnt/beta-catenin and Notch signaling pathways^[^^55^^,^^56^^]^. Consistently, our results emphasize that aggressive signature and metastatic capability of ESCC is inextricably linked to the crosstalk of Wnt/Notch signaling cascades with activation of the downstream target genes, such as *MMP-21, HLA-G,* and *IL-10*. Undoubtedly, future molecular investigations are required to clarify the biological interactions among these molecules.

Taken together, our study represents the strong regulatory axis describing crosstalk between *INPP5A*, *HLAG-1, IL-10,* and *MMP-21* markers in ESCC development. Notably, the combined detection of *INPP5A, HLA-G1, IL-10,* and *MMP-21* may serve as promising predictor markers for diagnostic in ESCC patients with relatively high sensitivity and specificity. Accordingly, our findings offer a novel insight into the mechanism of immune evasion mediated by the *INPP5A*/*HLAG-1*/*IL-10*/*MMP-21* regulatory network in the disease. 

## DECLARATIONS

### Acknowledgments

The authors gratefully acknowledge colleagues at the Human Genetics Division of Avicenna Research Institute (Tehran, Iran) for their scientific and technical supports.

### Ethical statement

The study protocol was approved by the Ethics Committee of the Mashhad University MUMS, Mashhad, Iran (ethical code: 88098). An appropriate signed informed consent document was achieved from each ESCC patient according to the 1964 Declaration of Helsinki.

### Data availability

The datasets used and/or analyzed during the current study are available from the corresponding author on reasonable request.

### Author contributions

SAK, performed the experiments presented in this manuscript and wrote the manuscript; AA, performed the experiments presented in this manuscript; MRA, revised the manuscript; MMF, conceptualized the study, analyzed the data, and critically revised the manuscript. All of the authors read and approved the manuscript. 

### Conflict of interest

None declared.

### Funding/support

The authors declare that no funds, grants, or other support were received during the preparation of this manuscript. 
